# Advanced maternal age as a risk factor for endometrial receptivity in repeated implantation failure: intersectional transcriptomic analysis and gene validation

**DOI:** 10.3389/fcell.2026.1904991

**Published:** 2026-08-26

**Authors:** Yaping Guo, Mianqiu Zhang, Zhenggang Wu, Xiaojing Hou, Juan Zhang, Xiaoxuan Yang, Hui Ding, Chenbo Ji, Xiufeng Ling, Yan Su, Rong Shen

**Affiliations:** 1 Nanjing Women and Children’s Healthcare Hospital, Women’s Hospital of Nanjing Medical University, Nanjing, China; 2 Nanjing Medical Key Laboratory of Female Fertility Preservation and Restoration, Nanjing, China

**Keywords:** advanced maternal age, endometrial receptivity test, endometrium, repeated implantation failure, RNA sequencing

## Abstract

**Background:**

Repeated implantation failure (RIF) remains a significant challenge in assisted reproductive technology. Endometrial receptivity (ER) plays a critical role in embryo implantation. However, the impact of advanced maternal age (AMA) on ER in this population remains unclear. The study aimed to evaluate whether maternal age is associated with displacement of the window of implantation (WOI) and explore potential endometrial transcriptomic characteristics.

**Methods:**

This retrospective observational study included 254 women with RIF who underwent evaluation of ER between January 2020 and March 2024. ER status and WOI timing were determined using a transcriptome-based receptivity assessment. Clinical characteristics were compared between women with normal receptivity and those with a 2-day pre-receptive endometrium. Endometrial biopsies were analyzed by RNA sequencing to identify differential gene expression associated with age and receptivity status. An independent small validation cohort was used for qRT-PCR validation.

**Results:**

Women in the impaired receptivity group (2-day pre-receptive) were significantly older than those in the normal receptivity group (34.3 ± 3.1 vs. 33.0 ± 4.1, *p* = 0.016). Multivariate logistic regression further demonstrated maternal age as an independent risk factor for ER displacement (OR = 1.118, 95% CI: 1.013–1.234, *p* = 0.027). Transcriptomic analysis identified age- and receptivity-associated transcriptomic changes, with 10 overlapping differentially expressed genes (DEGs) between the two comparisons, which were further examined by qRT-PCR in a small independent cohort (n = 10). As a result, four genes, including activating transcription factor 3 (*ATF3*), C-X-C motif chemokine ligand 1 (*CXCL1*), parathyroid hormone-like hormone (*PTHLH*), and non-specific cytotoxic cell receptor protein 1 (*NCCRP1*), were significantly downregulated in the AMA-RIF group compared with controls.

**Conclusion:**

AMA was independently associated with an increased likelihood of WOI displacement in women with RIF, suggesting that aberrant endometrial timing may be associated with implantation failure in this population. These findings provide a rationale for further investigation of personalized embryo transfer strategies in older patients, while the identified candidate genes should be regarded as preliminary molecular associations requiring further functional validation.

## Introduction

With the increasing trend of delayed childbearing, more women of advanced maternal age (AMA, >35 years) seek pregnancy through *in vitro* fertilization and embryo transfer (IVF–ET) (Ubaldi et al., 2019). To date, the success rate of IVF–ET has reached approximately 60% (Margalioth et al., 2006); however, 5%–10% of patients still experience repeated implantation failure (RIF) despite the transfer of morphologically high-quality embryos.

There is no universally accepted definition of RIF. In this study, RIF was defined as the failure of at least two embryo transfer (ET) cycles, each involving the transfer of one or more morphologically high-quality embryos (Busnelli et al., 2021). In women with RIF, implantation may repeatedly fail even when high-quality embryos are transferred, suggesting that endometrial factors and impaired embryo-endometrium crosstalk contribute significantly to implantation failure (Sehring et al., 2022; Neves et al., 2019; Hiraoka et al., 2023; Norwitz et al., 2001). Shapiro et al. (2016) found that patients older than 35 years exhibited lower implantation and live birth rates in IVF cycles. Given the considerable physical and psychological burden associated with RIF, earlier diagnosis and intervention have increasingly been advocated in clinical practice (Ma et al., 2022).

The window of implantation (WOI), typically occurring between days 20 and 24 of a 28-day menstrual cycle, represents the period when the endometrium is optimally receptive to embryo implantation (Achache and Revel, 2006; Kliman and Frankfurter, 2019). In IVF–ET cycles using hormone replacement therapy (HRT), the fifth day after the initiation of progesterone administration (P+5) generally corresponds to the WOI for blastocyst transfer (Gomez et al., 2015; Mumusoglu et al., 2021). In patients with RIF, this critical period may be temporally displaced or molecularly dysregulated, thereby contributing to implantation failure. Abnormal endometrial receptivity (ER) has been implicated in up to two-thirds of RIF cases (Guo et al., 2023). Emerging evidence suggests that AMA not only impairs ovarian reserve and embryonic competence but may also alter ER through endocrine, immune, and transcriptomic mechanisms (Devesa-Peiro et al., 2022).

Historically, Noyes et al. (2019) first introduced histological criteria for evaluating ER in the 1950s. Since then, transcriptomics profiling has enabled more precise molecular characterization of endometrial status. RNA sequencing combined with machine learning approaches has been increasingly applied to infer the WOI based on endometrial gene expression patterns, providing a temporal framework for characterizing receptivity-related molecular features rather than directly measuring ER itself (Diaz-Gimeno et al., 2011; Ruiz-A and lonso, 2021; He, 2021). However, evidence regarding the clinical utility of receptivity-guided strategies remains inconsistent. While several studies have suggested no significant improvement in pregnancy outcomes among unselected populations with RIF (Fodina et al., 2021; Saxtorph et al., 2020), others have reported improved implantation and live birth rates in patients with RIF (Xu, 2025; Ohara et al., 2022).

Alterations in ER are closely linked to dynamic changes in gene expression (Bashiri et al., 2018). Beyond advancing our understanding of the implantation process, transcriptomic features associated with receptivity may also provide potential therapeutic targets for implantation failure (Diaz-Gimeno et al., 2014). Although maternal aging is well known to impair oocyte quality and embryonic competence, its impact on ER remains incompletely understood. Therefore, the present study aimed to investigate whether advanced maternal age is associated with displacement of the WOI and explore potential transcriptomic characteristics of the endometrium in women with RIF.

## Methods

### Study population

**FIGURE 1:**
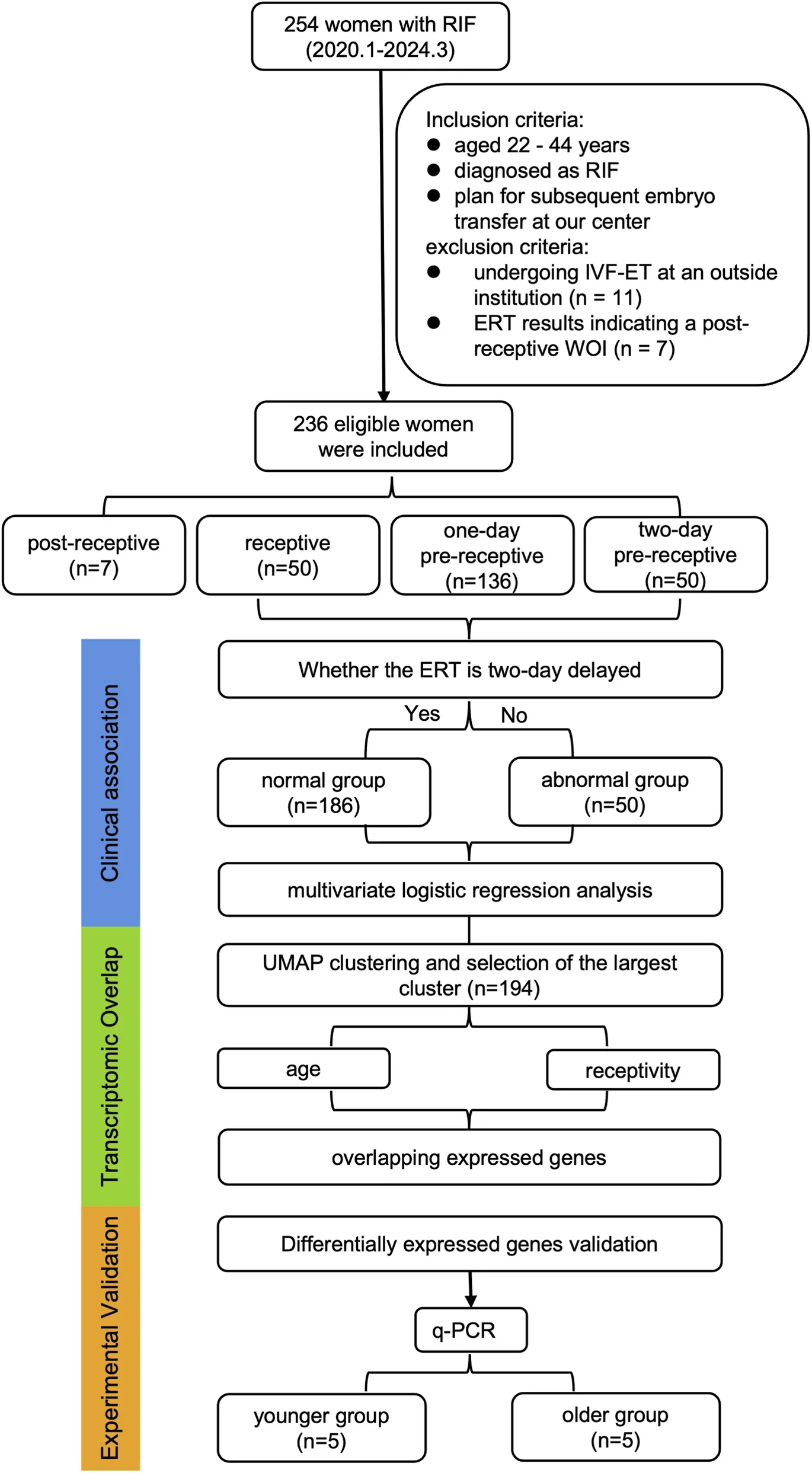
Flow chart.

This retrospective observational study analyzed clinical data from 254 women diagnosed with RIF who were treated at the Department of Reproductive Medicine in Nanjing Women and Children’s Healthcare Hospital from January 2020 to March 2024. Inclusion criteria were age between 22 and 44 years and a planned subsequent ET at our center. Exclusion criteria included (i) undergoing IVF–ET at an outside institution (n = 11) and (ii) transcriptomic analysis indicating a post-receptive WOI (n = 7), due to insufficient sample size for meaningful analysis. After applying these criteria, a total of 236 eligible participants were included in the study. Considering ER represents a dynamic temporal continuum and that a 1-day pre-receptive endometrium may reflect a transitional physiological state, we defined receptive and 1-day pre-receptive samples as normal ER, whereas 2-day pre-receptive samples were classified as abnormal ER (Figure 1).

The study was approved by the Ethics Committee of Nanjing Women and Children’s Healthcare Hospital (ethics number [2020] YL-009), and all participants signed the informed consent form. Endometrial biopsy and transcriptomic analysis were performed once for each participant. Clinical data were retrieved from the hospital information management system. This study was conducted in accordance with the Declaration of Helsinki.

#### Endometrial preparation and biopsy

All patients received a standardized HRT regimen for endometrial preparation. In brief, beginning on the second day of the menstrual cycle, patients received 4 or 6 mg of estradiol valerate daily (Progynova, Bayer, France). The dosage was subsequently adjusted based on endometrial thickness (EMT) and serum estradiol (E2) and progesterone (P) levels, typically reaching 6–10 mg per day after 1 week.

Luteal phase support (LPS) was initiated when the EMT reached 7 mm, the serum E2 level was ≥200 pg/ml, and the serum P level was <1.5 ng/ml (P+0). Endometrial biopsies were performed on P+5 using a disposable endometrial sampler (Jiangxi Nuode Medical Device Co., Ltd., Jiangxi, China). Biopsied tissues (≥ 8 mm^3^) were transferred to cryotubes containing sample preservation solution (Yikon, China) and immediately stored at −20 °C for RNA sequencing.

#### RNA sequencing

Total RNA was extracted from endometrial tissues and quantified using the Qubit RNA HS assay. cDNA synthesis and amplification were performed using a low-input RNA amplification kit, followed by library preparation and sequencing on the Illumina NextSeq 550 platform.

ER status and WOI timing were assessed using a validated transcriptome-based machine learning model developed by Yikon Genomics in collaboration with Xiangya Hospital of Central South University. The model integrates gene expression signatures of receptivity-associated genes to classify samples as pre-receptive, receptive, or post-receptive. Transcriptomic profiles were mapped to a reference framework to estimate temporal displacement from the optimal WOI.

Bioinformatic processing and normalization were performed using the ChromGo analysis platform. Read counts were normalized using the geometric median ratio method implemented in the DESeq2 R package (DESeq2_1.50.2).

To evaluate transcriptomic heterogeneity and ensure data quality, unsupervised dimensionality reduction using Uniform Manifold Approximation and Projection (UMAP) was performed on normalized gene expression data as an exploratory quality-control step. Sample-level clustering was used to identify potential outliers and transcriptionally distinct subgroups. This analysis revealed three clusters, including one small and clearly separated cluster. Based on predefined analytic criteria, downstream analyses were restricted to the largest cluster (n = 194), representing the dominant transcriptional structure of the dataset and serving as the primary analytic cohort. This step was performed prior to differential expression analysis and was not guided by clinical phenotypes or outcome variables.

#### Identification and functional annotation of the differentially expressed genes

Differentially expressed genes (DEGs) were identified using the DESeq2 package with an adjusted *p*-value <0.05 and |log_2_fold change| ≥1. Functional enrichment analysis of DEGs was performed using the clusterProfiler package (clusterProfiler v4.18.1) to identify biological pathways associated with age and ER status. Differential expression analyses were conducted separately for maternal age groups and ER status, and overlapping genes between the two DEG sets were identified.

#### qRT-PCR validation

Total RNA from endometrial samples was extracted using TRIzol reagent. Reverse transcription was performed to generate cDNA, and quantitative real-time PCR was conducted using SYBR Green chemistry on a ViiA™ 7 Real-Time PCR system. GAPDH served as the internal control. Primer sequences are provided in Supplementary Table S1.

#### Statistical analysis

Continuous variables were expressed as the mean ± standard deviation for normally distributed data and as the median (interquartile range) for non-normally distributed data. Normally distributed variables were compared using the independent samples t-test, whereas non-normally distributed variables were analyzed using the Mann–Whitney U test. Categorical variables were compared using the chi-square test or Fisher’s exact test, as appropriate. Multivariate logistic regression analysis was performed to identify independent risk factors for impaired ER. Variables with statistical significance in univariate analysis (*p* < 0.1) were initially screened. In addition, clinically relevant variables were also included in the multivariate logistic regression model. Odds ratios (ORs) and 95% confidence intervals (CIs) were calculated to evaluate associations between maternal age and ER status.

Differential expression analysis was performed using DESeq2, and *p*-values were adjusted using the Benjamini–Hochberg method to control the false discovery rate. A two-sided *p* < 0.05 was considered statistically significant.

## Results

### Clinical characteristics and receptivity status in RIF patients

A total of 236 women with RIF were included in the final analysis based on transcriptome-derived ER assessment. Among them, 50 (21.2%) exhibited a receptive endometrium at P+5. The remaining 186 patients showed displaced receptivity, including 136 (57.6%) classified as 1-day pre-receptive and 50 (21.2%) classified as 2-day pre-receptive (Supplementary Figure S1).

For clinical comparison, patients with receptive or 1-day pre-receptive endometrium were grouped as normal ER, while those with 2-day pre-receptive endometrium were classified as abnormal ER (Table 1).

**TABLE 1:** Baseline of RIF patients with different receptivity status.

Term	Normal	Abnormal	*p*
	n = 186	n = 50	
Age^a^	33.0 ± 4.1	34.3 ± 3.1	0.016
Body mass index^a^	22.2 ± 3.0	22.2 ± 2.8	0.880
Infertile type^c^	​	​	0.727
Primary	87 (46.8)	22 (44.0)	-
Secondary	99 (53.2)	28 (56.0)	-
bFSH^a^	8.0 ± 5.2	7.5 ± 2.7	0.471
bLH^a^	5.2 ± 4.0	4.3 ± 2.9	0.129
Basal estradiol^a^	47.6 ± 44.3	43.8 ± 22.0	0.554
Parity^b^	0 (0–0)	0 (0–0)	-
AMH^b^	3.8 (2.1–6.2)	3.8 (1.8–6.6)	0.777
Endometrial thickness^a^	8.4 ± 1.4	8.8 ± 2.0	0.255
Scarred uterus^c^	21 (11.4)	6 (12.0)	0.898
Prior history of endometritis^c^	43 (23.1)	9 (18.0)	0.438
Hydrosalpinx^c,^ *	5 (2.7)	1 (2.0)	1.000
Mild uterine adhesions^c^	45 (24.2)	11 (22.0)	0.746
Endometriosis^c,^ *	9 (4.8)	3 (6.0)	0.721
PCOS^c,^ *	11 (5.9)	2 (4.0)	1.000
Immune abnormality^c^	21 (11.3)	4 (8.0)	0.502

a: Data are expressed as the mean ± SD for continuous variables following a normal distribution. b: Data are expressed as the median (first quartile, third quartile) for continuous variables not normally distributed. c: Data are expressed as the numbers (percentages) for categorical variables. * Fisher’s exact test.

bFSH, basal follicle-stimulating hormone; bLH, basal luteinizing hormone; AMH, anti-Müllerian hormone; PCOS, polycystic ovarian syndrome.

Women in the abnormal ER group were significantly older than those in the normal ER group (34.3 ± 3.1 vs. 33.0 ± 4.1 years, *p* = 0.016). No significant differences were observed in BMI, infertility type, baseline reproductive hormones (FSH, LH, and E2), AMH levels, parity, EMT on progesterone initiation day (P+0), or comorbid gynecological and immune conditions (all *p* > 0.05).

Multivariate logistic regression analysis using ER displacement (2-day shift vs. non-shift) as the dependent variable identified maternal age as an independent predictor of ER displacement (*p* = 0.027) (Table 2).

**TABLE 2:** Multivariate logistic regression analysis.

Variable	OR (95% CI)	*p*	Multivariate analysis
Age	1.118 (1.013–1.234)	0.027	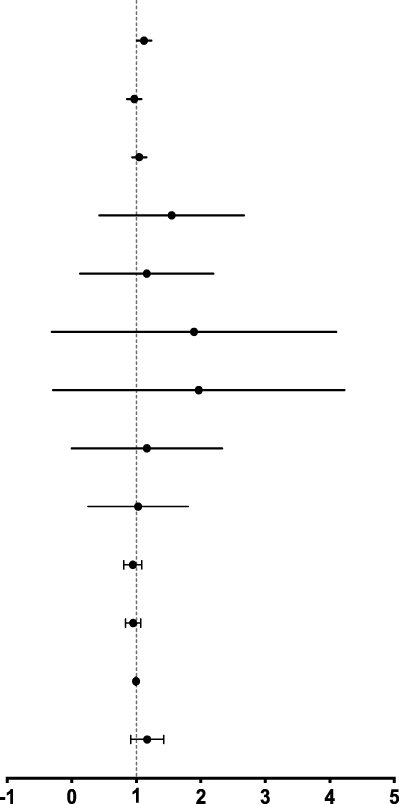
Body mass index	0.965 (0.861–1.082)	0.545	
anti-Müllerian hormone	1.042 (0.939–1.156)	0.440	
Infertile type	1.279 (0.588–2.780)	0.535	
Parity	0.856 (0.317–2.313)	0.759	
Scarred uterus	1.043 (0.247–4.401)	0.954	
Endometriosis	1.107 (0.270–4.533)	0.888	
Immune abnormality	0.778 (0.244–2.474)	0.670	
Mild uterine adhesions	0.833 (0.368–1.884)	0.660	
bFSH	0.939 (0.811–1.088)	0.402	
bLH	0.946 (0.835–1.071)	0.379	
bE_2_	0.997 (0.987–1.008)	0.620	
EMT	1.151 (0.924–1.433)	0.211	

Whether the ERT is 2-day displaced; AMH, anti-Müllerian hormone; PCOS, polycystic ovarian syndrome; EMT, endometrial thickness.

### Implantation outcomes between young and aged groups following personalized embryo transfer

We further analyzed the first personalized embryo transfer (pET) cycles following ER assessment. A total of 51 women underwent pET with the transfer of at least one high-quality embryo, including 21 patients in the young group (≤30 years) and 30 in the aged group (>35 years).

After adjustment of embryo quality and WOI timing, implantation outcomes were comparable between the two groups. The clinical pregnancy rate was 47.6% in the young group and 40.0% in the aged group (*p* = 0.649), while the implantation rate was 34.3% and 27.9%, respectively (*p* = 0.568) (Supplementary Table S2).

### Transcriptomic signatures associated with maternal age

As described in the Methods, unsupervised dimensionality reduction using UMAP was performed as an exploratory quality-control assessment of the RNA-seq data. Three clusters were identified, including one small cluster that was clearly separated from the main sample population. According to the predefined analytic strategy, downstream analyses were performed using the largest and most transcriptionally homogeneous cluster (n = 194) (Supplementary Figure S2).

**FIGURE 2:**
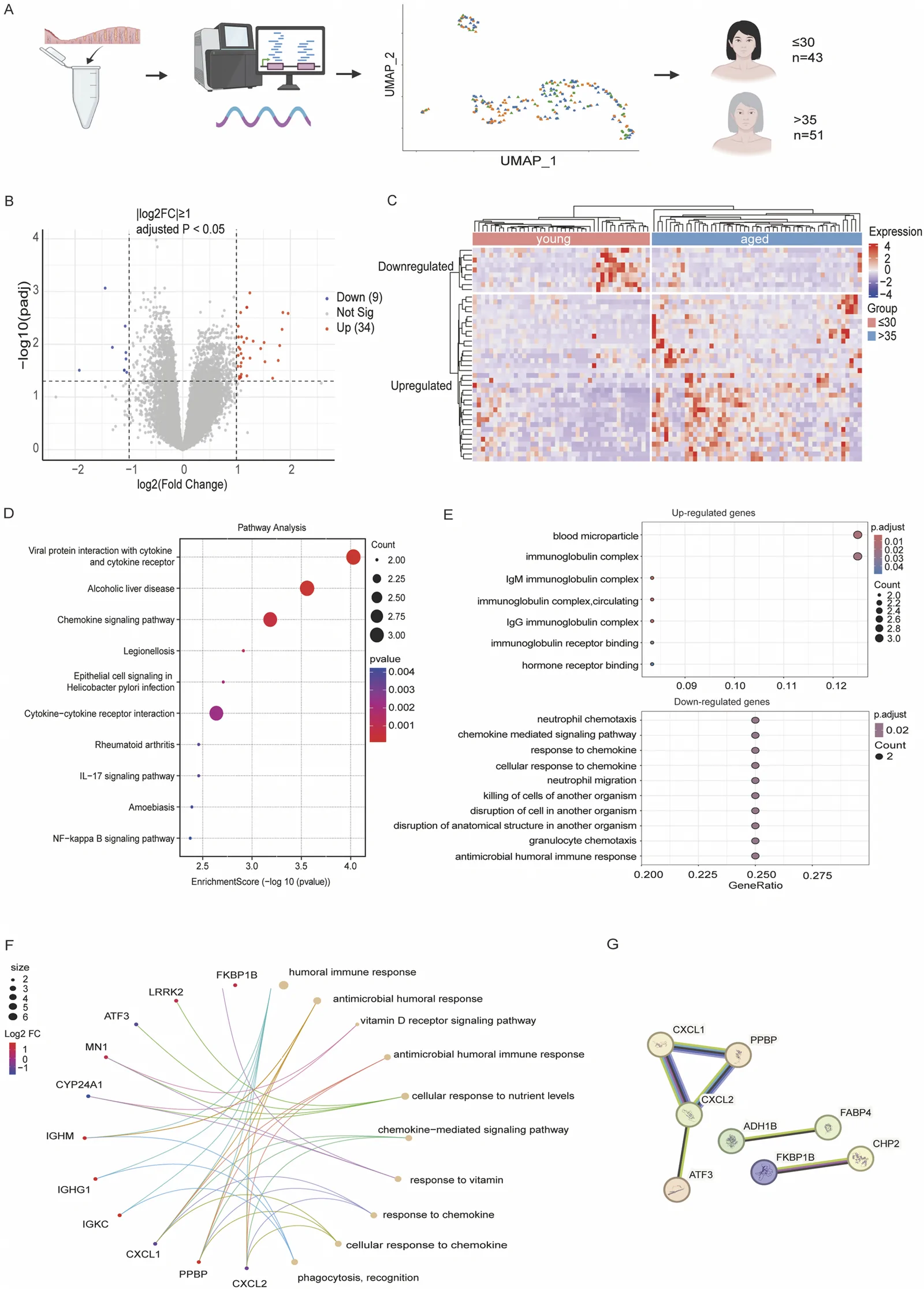
Differential transcriptomic landscape of the endometrium in young (≤30 years, n = 43) and aged (>35 years, n = 51) women with RIF. **(A)** Schematic diagram of the analysis. **(B)** Volcano plot showing DEGs between the endometrial tissues of women in the young and aged groups (adjusted *p*-value <0.05 and |log_2_FC| ≥1). **(C)** Heatmap of the DEGs based on hierarchical clustering. **(D)** Gene Ontology enrichment analysis of DEGs based on the identified genes. **(E)** KEGG pathway enrichment analysis of DEGs based on the identified genes. **(F)** Cnetplot visualization depicting the relationship between DEGs and their associated enriched GO or KEGG terms. **(G)** Protein–protein interaction network constructed using the STRING database for DEGs.

The age threshold of 35 years was selected because it is the conventional clinical definition of AMA and has been widely adopted. Recent population-based evidence further supports its relevance to differences in prenatal care utilization and perinatal outcomes (Geiger et al., 2021). To achieve a clearer comparison between distinct age groups, women aged 31–35 years were excluded from the age-stratified differential expression analysis. Differential expression analysis was performed between the young group (≤30 years, n = 43) and the aged group (>35 years, n = 51) (Figure 2A). A total of 43 differentially expressed genes (DEGs) were identified in the aged group, including 34 upregulated and 9 downregulated genes (adjusted *p* < 0.05, |log_2_FC| ≥1) (Figure 2B; Supplementary Data Sheet 1).

Hierarchical clustering based on these DEGs showed clear separation between the two age groups (Figure 2C). Pathway enrichment analysis may indicate significant involvement of immune-related pathways. KEGG analysis revealed enrichment in cytokine–cytokine receptor interaction, chemokine signaling pathway, viral protein interaction with cytokine and cytokine receptor, and alcoholic liver disease pathways (Figure 2D).

Gene Ontology analysis showed that upregulated genes in the aged group were enriched in blood microparticles and immunoglobulin complexes, whereas downregulated genes were enriched in chemokine-mediated signaling, neutrophil chemotaxis, and cellular response to chemokine stimulus (Figure 2E).

Network analysis further highlighted key regulatory relationships among these genes. Cnetplot analysis revealed central hub genes connecting multiple enriched pathways (Figure 2F), and STRING-based protein-protein interaction analysis demonstrated a tightly connected gene interaction module (Figure 2G).

### Transcriptomic signatures associated with ER displacement

**FIGURE 3:**
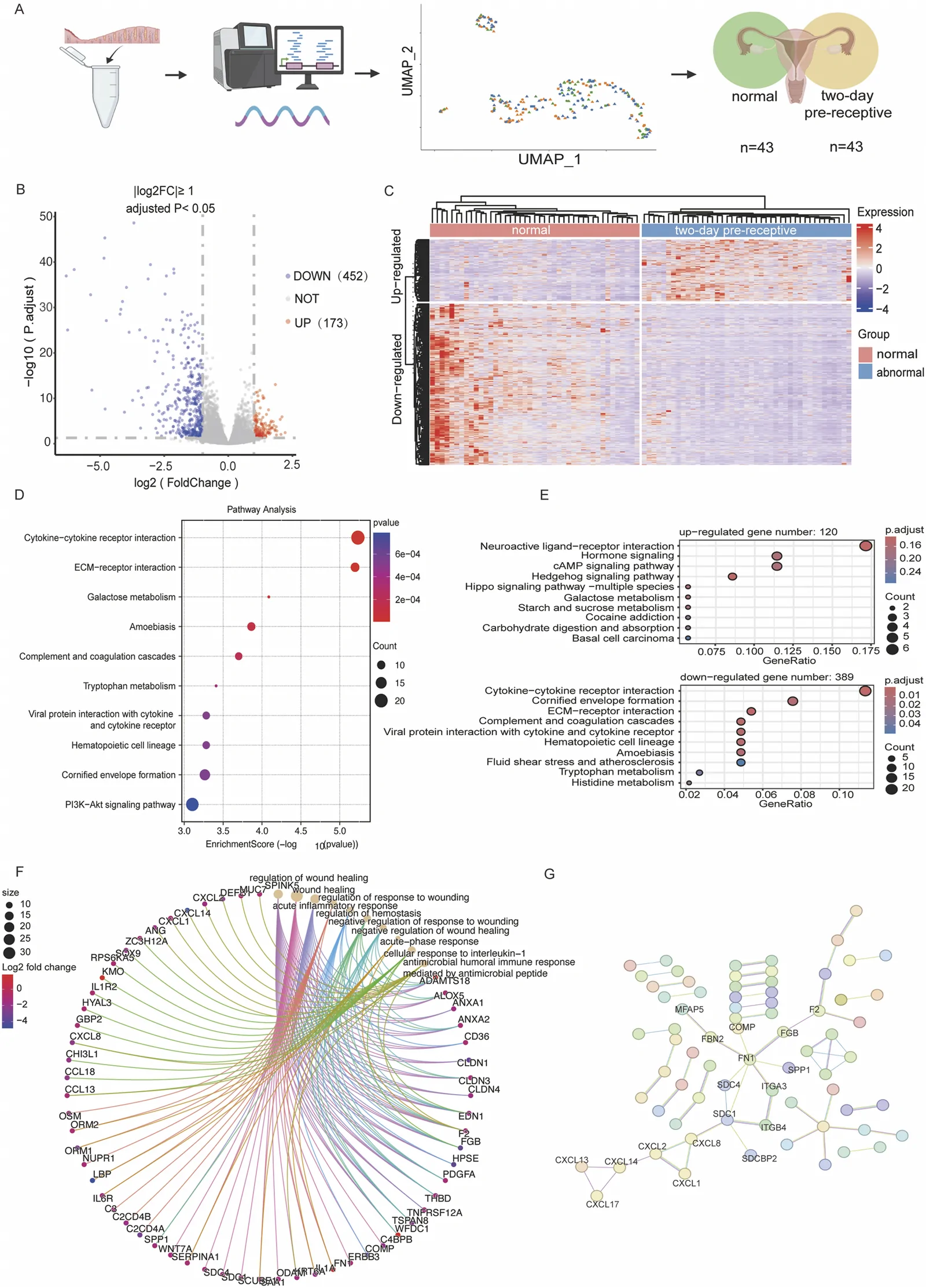
Differential transcriptomic landscape of the endometrium in women with normal receptivity (n = 43) and 2-day displaced receptivity (n = 43). **(A)** Schematic diagram of the analysis. **(B)** Volcano plot showing DEGs between the endometrial tissues of women in 2-day displaced and normal receptivity groups (adjusted *p*-value <0.05 and |log_2_FC| ≥1). **(C)** Heatmap of the DEGs based on hierarchical clustering. **(D)** Gene Ontology enrichment analysis of DEGs based on the identified genes. **(E)** KEGG pathway enrichment analysis of DEGs based on the identified genes. **(F)** Cnetplot visualization depicting the relationship between DEGs and their associated enriched GO or KEGG terms. **(G)** Protein–protein interaction network constructed using the STRING database for DEGs.

To investigate molecular features associated with abnormal receptivity, transcriptomic profiles of women with 2-day displaced ER (n = 43) were compared with those with normal ER (n = 43) (Figure 3A).

Differential expression analysis identified a distinct set of DEGs between the two groups (Figure 3B; Supplementary Data Sheet 2; Figure 3C). KEGG enrichment analysis revealed significant involvement of pathways including cytokine–cytokine receptor interaction, ECM–receptor interaction, PI3K–AKT signaling, and viral protein interaction with cytokine and cytokine receptor (Figure 3D).

GO analysis may indicate that downregulated genes in the displaced ER group were associated with cytokine–cytokine receptor interaction, cornified envelope formation, and ECM–receptor interaction, whereas upregulated genes were enriched in neuroactive ligand–receptor interaction, hormone signaling, and cAMP signaling pathways (Figure 3E).

Network visualization using Cnetplot identified hub genes with strong pathway connectivity (Figure 3F), and STRING-based analysis demonstrated a distinct interaction network among the identified DEGs (Figure 3G).

### Shared transcriptomic signatures between maternal age and ER displacement

**FIGURE 4:**
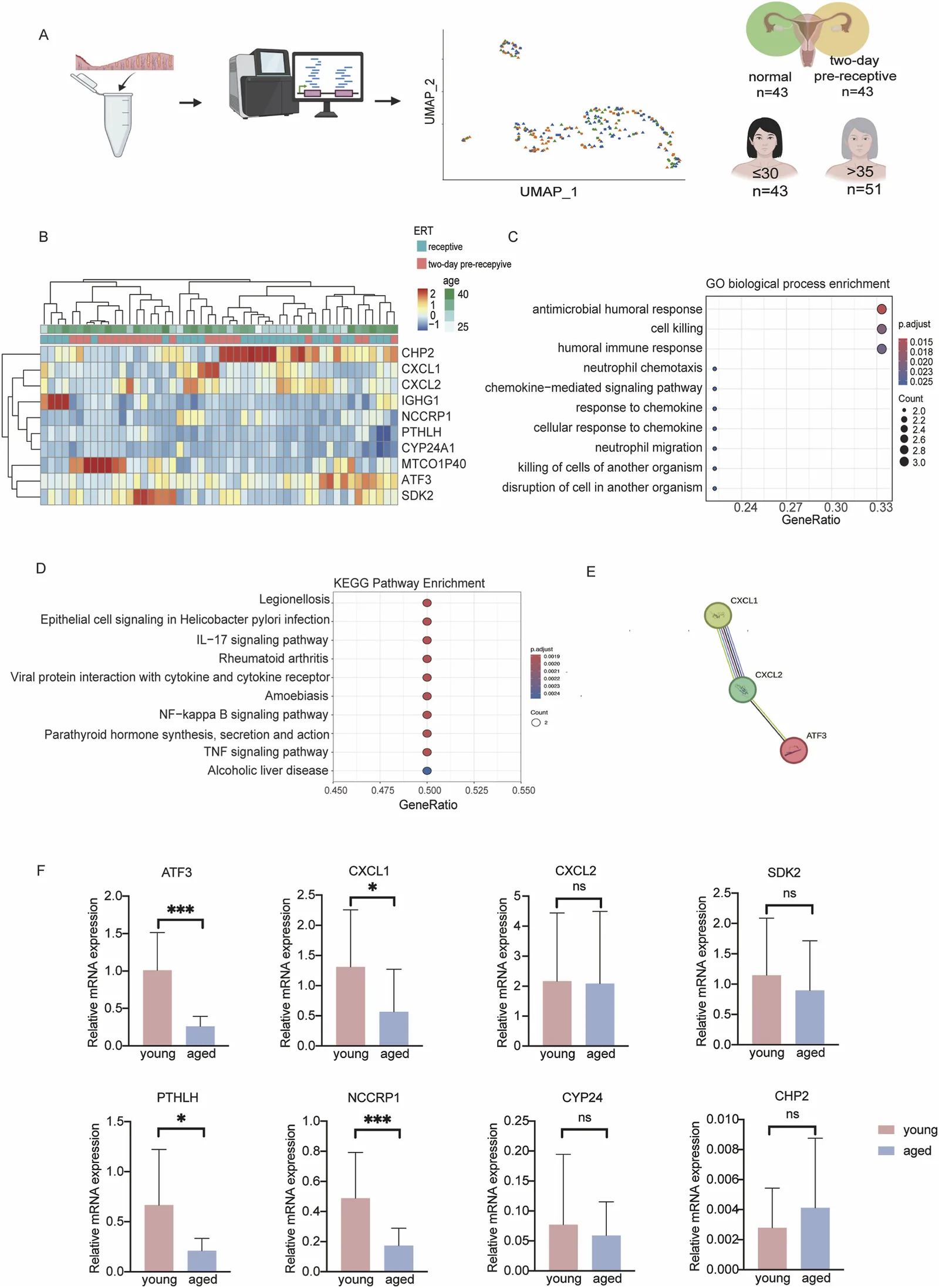
Overlapping transcriptomic landscape of the endometrium between age and receptivity of women with RIF. **(A)** Schematic diagram of the analysis. **(B)** Heatmap of the overlapping DEGs based on hierarchical clustering. **(C)** Gene Ontology enrichment analysis of overlapping DEGs based on the intersected gene set. **(D)** KEGG pathway enrichment analysis of overlapped DEGs based on the intersected gene set. **(E)** Protein–protein interaction network constructed using the STRING database for overlapped DEGs. **(F)** Validation of overlapped DEGs by qRT-PCR in an independent cohort (young, n = 5; aged, n = 5). Given the limited number of overlapping DEGs (n = 10), enrichment analyses should be considered exploratory and interpreted with caution. The qRT-PCR validation results should be interpreted cautiously due to the limited sample size.

To identify genes potentially linking maternal age and altered ER, DEGs identified from the age and ER comparisons were intersected. This analysis identified 10 overlapping genes, namely, *ATF3*, *CXCL1*, *CXCL2*, *IGHG1*, *NCCRP1*, *PTHLH*, *CYP24A1*, *MTCO1P40*, *CHP2*, and *SDK2* (Figures 4A,B).

Functional annotation suggested that these genes were associated with immune-related biological processes, including cell killing and humoral immune response (Figure 4C). KEGG pathway analysis indicated enrichment in IL-17 signaling, TNF signaling, NF-κB signaling, and cytokine–cytokine receptor interaction pathways (Figure 4D). Protein–protein interaction analysis suggested potential interactions among *CXCL1*, *CXCL2*, and *ATF3* (Figure 4E).

To validate these findings, qRT-PCR was performed using an independent cohort of endometrial samples (n = 5 per group). *MTCO1P40* was excluded from qRT-PCR validation because it is a pseudogene, and *IGHG1* was not selected because its expression mainly represents endometrial immune-cell infiltration rather than intrinsic endometrial cellular responses. Consistent with RNA-seq results, *ATF3*, *CXCL1*, *PTHLH*, and *NCCRP1* were significantly downregulated in the aged group (Figure 4F).

## Discussion

With the increasing trend of delayed childbearing, the proportion of women of AMA undergoing IVF–ET has grown significantly. Notably, a growing subset of these women experience RIF, highlighting the need to explore potential age-related impairments in ER (Bastu et al., 2019; Garcia et al., 2018; Li et al., 2025; Attali and Yogev, 2021). Although previous studies have primarily focused on aging-associated defects in oocytes and embryos, the role of endometrial aging remains incompletely understood and controversial (Pathare et al., 2023). Endometrial factors are estimated to account for about two-thirds of the causes of embryo implantation failure (Craciunas et al., 2019). A randomized controlled trial has reported that ER-guided pET may be associated with improved implantation and live birth rates in women of advanced maternal age with RIF (Barbakadze, 2024). In this study, integrating transcriptomic profiling with WOI-based receptivity assessment, we observed that women with RIF and AMA exhibited distinct endometrial transcriptomic features during the window of implantation.

When comparing patients with or without marked ER displacement, women with abnormal receptivity were significantly older (*P* = 0.016). Multivariate regression analysis further confirmed that age was independently associated with ER status (OR: 1.118, 95% CI: 1.013-1.234, *P* = 0.027). Consistent with our findings, previous studies have found that advanced-age women are more likely to exhibit WOI displacement (Fujii and Oguchi, 2023; Zhao, 2023; Yaron et al., 1993).

Transcriptomic analysis revealed distinct gene expression patterns between aged and younger groups. Functional enrichment analyses may indicate dysregulation of pathways related to cytokine signaling and immune regulation. In particular, genes involved in chemokine-mediated signaling, neutrophil chemotaxis, and cellular responses to chemokines were downregulated in the aged endometrium. These findings suggest that maternal aging may alter the endometrial immune microenvironment, potentially impairing appropriate responses to embryonic signals during implantation.

Similarly, patients with displaced ER exhibited enrichment of immune-related pathways among downregulated genes. Immune regulation plays a pivotal role in regulating uterine receptivity (Robertson et al., 2022). Such alterations may be linked with the establishment of a receptive micro-environment for embryo-endometrial communication. In the proteomic analysis of endometrial samples from women with RIF and controls, GO analysis likewise showed enrichment in terms related to the immune response (Wang et al., 2021). Shi et al. (2018) reported that in transcriptomic profiling of endometrial samples from women with RIF during the WOI, the downregulated mRNAs were likewise enriched in the pathways “*cytokine–cytokine receptor interaction*” and “*ECM–receptor interaction.*”

To explore shared molecular signatures between AMA and displaced ER, we identified 10 overlapping DEGs. Functional annotation of this limited gene set suggested involvement in immune-related processes, such as neutrophil chemotaxis and humoral responses. However, given the small number of genes, these enrichment results should be interpreted cautiously and considered exploratory. Notably, several of these genes have previously been implicated in implantation biology. Boespflug et al. found that *ATF3* is a regulator of mouse neutrophil migration (Boespflug et al., 2014). Cheng et al. (2017) also reported a crucial role for *ATF3* in enhancing ER and embryo attachment via upregulation of leukemia inhibitory factor (LIF). *CXCL1*, a small cytokine of the CXC chemokine family, is known to promote endothelial cell proliferation, migration, and tube formation, critical steps in angiogenesis (Baston-Buest et al., 2017). Its role in decidual angiogenesis has been demonstrated both *in vitro* and *in vivo* (Ma et al., 2021; Izumi et al., 2015; Baston-Bust et al., 2013), and its downregulation, along with *CXCL2*, may impair immune cell recruitment and maternal vascular remodeling essential for implantation. Transcriptomic analyses have shown significant downregulation of *CXCL1* and *C4BPA* in RIF patients with a thin endometrium (Kurmanova et al., 2024). *PTHLH*, which is important in early pregnancy, when antagonized, induces fetoplacental growth restriction through mitochondrial apoptosis and decreased platelet endothelial cell adhesion molecule expression (Thota et al., 2005). *NCCRP1*, associated with cell proliferation, has been detected in the ER profile in recurrent miscarriage (Craciunas, 2021). Such displacement may reflect inadequate or delayed decidualization. Validation in an independent cohort confirmed differential expression of four genes, providing preliminary support for their involvement in receptivity alterations.

Several limitations should be acknowledged. First, this was a retrospective single-center study, and potential selection bias cannot be excluded. Second, the validation cohort was relatively small, and larger studies are needed to confirm these findings. Third, the observational design limits causal inference between AMA and ER displacement. In addition, the grouping strategy for ER may not fully capture the continuous nature of the WOI, and alternative classification strategies should be explored in future studies. Further mechanistic studies are required to clarify how maternal aging affects endometrial function at the molecular level.

Our study identified several candidate genes associated with age-related transcriptomic alterations in the endometrium. These findings provide preliminary molecular evidence of immune-related transcriptomic changes in the endometrium of women with AMA and RIF. Further studies with larger cohorts and functional investigations are warranted to elucidate the biological roles of these candidate genes in endometrial aging and reproductive outcomes.

## Data Availability

The datasets generated and analyzed during the current study are available in the OMIX repository under accession number OMIX013026. The dataset is subject to controlled access.

## Supplementary material

Supplementary Figure S1 Distribution of endometrial receptivity among 236 women with RIF. Among the 236 RIF patients who underwent ERT, 50 (21.2%) were classified as receptive. Of the remaining 186 patients with a displaced WOI, 136 (57.6%) were classified as 1-day pre-receptive and 50 (21.2%) were classified as 2-day pre-receptive.

Supplementary Figure S2 Global transcriptomic landscape stratified by age and ER status via UMAP clustering. Unsupervised dimensionality reduction using UMAP was performed on the transcriptomic data from the primary analytic cohort (n = 194) to visualize global expression patterns across age groups and ER status. Three distinct clusters were identified.

## References

[B1] Achache H. Revel A. (2006). Endometrial receptivity markers, the journey to successful embryo implantation. Hum. Reprod. Update 12 (6), 731–746. 10.1093/humupd/dml004 16982667

[B2] Attali E. Yogev Y. (2021). The impact of advanced maternal age on pregnancy outcome. Best. Pract. Res. Clin. Obstet. Gynaecol. 70, 2–9. 10.1016/j.bpobgyn.2020.06.006 32773291

[B3] Barbakadze T. (2024). Assessment of the role of endometrial receptivity analysis in enhancing assisted reproductive technology outcomes for advanced-age patients. Cureus 16 (6), e62949. 10.7759/cureus.62949 39044886 PMC11264563

[B4] Bashiri A. Halper K. I. Orvieto R. (2018). Recurrent implantation Failure-update overview on etiology, diagnosis, treatment and future directions. Reprod. Biol. Endocrinol. 16 (1), 121. 10.1186/s12958-018-0414-2 30518389 PMC6282265

[B5] Baston-Buest D. M. Altergot-Ahmad O. Pour S. J. Krüssel J. S. Markert U. R. Fehm T. N. (2017). Syndecan-1 acts as an important regulator of CXCL1 expression and cellular interaction of human endometrial stromal and trophoblast cells. Mediat. Inflamm. 2017, 8379256. 10.1155/2017/8379256 28293067 PMC5331292

[B6] Baston-Bust D. M. Schanz A. Böddeker S. J. Altergot-Ahmad O. Krüssel J. S. Rein D. (2013). CXCL1 expression in human decidua *in vitro* is mediated *via* the MAPK signalling cascade. Cytokine 64 (1), 79–85. 10.1016/j.cyto.2013.07.023 23953856

[B7] Bastu E. Demiral I. Gunel T. Ulgen E. Gumusoglu E. Hosseini M. K. (2019). Potential marker pathways in the endometrium that may cause recurrent implantation failure. Reprod. Sci. 26 (7), 879–890. 10.1177/1933719118792104 30081718

[B8] Boespflug N. D. Kumar S. McAlees J. W. Phelan J. D. Grimes H. L. Hoebe K. (2014). ATF3 is a novel regulator of mouse neutrophil migration. Blood 123 (13), 2084–2093. 10.1182/blood-2013-06-510909 24470589 PMC3968391

[B9] Busnelli A. Somigliana E. Cirillo F. Baggiani A. Levi-Setti P. E. (2021). Efficacy of therapies and interventions for repeated embryo implantation failure: a systematic review and meta-analysis. Sci. Rep. 11 (1), 1747. 10.1038/s41598-021-81439-6 33462292 PMC7814130

[B10] Cheng X. Liu J. Shan H. Sun L. Huang C. Yan Q. (2017). Activating transcription factor 3 promotes embryo attachment *via* up-regulation of leukemia inhibitory factor *in vitro* . Reprod. Biol. Endocrinol. 15 (1), 42. 10.1186/s12958-017-0260-7 28577574 PMC5457579

[B11] Craciunas L. (2021). The transcriptomic profile of endometrial receptivity in recurrent miscarriage. Eur. J. Obstet. Gynecol. Reprod. Biol. 261, 211–216. 10.1016/j.ejogrb.2021.04.041 33971384

[B12] Craciunas L. Gallos I. Chu J. Bourne T. Quenby S. Brosens J. J. (2019). Conventional and modern markers of endometrial receptivity: a systematic review and meta-analysis. Hum. Reprod. Update 25 (2), 202–223. 10.1093/humupd/dmy044 30624659

[B13] Devesa-Peiro A. Sebastian-Leon P. Parraga-Leo A. Pellicer A. Diaz-Gimeno P. (2022). Breaking the ageing paradigm in endometrium: endometrial gene expression related to cilia and ageing hallmarks in women over 35 years. Hum. Reprod. 37 (4), 762–776. 10.1093/humrep/deac010 35085395

[B14] Diaz-Gimeno P. Horcajadas J. A. Martínez-Conejero J. A. Esteban F. J. Alamá P. Pellicer A. (2011). A genomic diagnostic tool for human endometrial receptivity based on the transcriptomic signature. Fertil. Steril. 95 (1), 50–60. 10.1016/j.fertnstert.2010.04.063 20619403

[B15] Diaz-Gimeno P. Ruíz-Alonso M. Blesa D. Simón C. (2014). Transcriptomics of the human endometrium. Int. J. Dev. Biol. 58 (2-4), 127–137. 10.1387/ijdb.130340pd 25023678

[B16] Fodina V. Dudorova A. Erenpreiss J. (2021). Evaluation of embryo aneuploidy (PGT-A) and endometrial receptivity (ERA) testing in patients with recurrent implantation failure in ICSI cycles. Gynecol. Endocrinol. 37 (Suppl. 1), 17–20. 10.1080/09513590.2021.2006466 34937515

[B17] Fujii S. Oguchi T. (2023). Age- and endometrial microbiota-related delay in development of endometrial receptivity. Reprod. Med. Biol. 22 (1), e12523. 10.1002/rmb2.12523 37383030 PMC10298046

[B18] Garcia D. Brazal S. Rodríguez A. Prat A. Vassena R. (2018). Knowledge of age-related fertility decline in women: a systematic review. Eur. J. Obstet. Gynecol. Reprod. Biol. 230, 109–118. 10.1016/j.ejogrb.2018.09.030 30248536

[B19] Geiger C. K. Clapp M. A. Cohen J. L. (2021). Association of prenatal care services, maternal morbidity, and perinatal mortality with the advanced maternal age cutoff of 35 years. JAMA Health Forum 2 (12), e214044. 10.1001/jamahealthforum.2021.4044 35977294 PMC8796879

[B20] Gomez E. Ruíz-Alonso M. Miravet J. Simón C. (2015). Human endometrial transcriptomics: implications for embryonic implantation. Cold Spring Harb. Perspect. Med. 5 (7), a022996. 10.1101/cshperspect.a022996 25818663 PMC4484960

[B21] Guo J. Zhou W. Sacco M. Downing P. Dimitriadis E. Zhao F. (2023). Using organoids to investigate human endometrial receptivity. Front. Endocrinol. (Lausanne) 14, 1158515. 10.3389/fendo.2023.1158515 37693361 PMC10484744

[B22] He A. (2021). The role of transcriptomic biomarkers of endometrial receptivity in personalized embryo transfer for patients with repeated implantation failure. J. Transl. Med. 19 (1), 176. 10.1186/s12967-021-02837-y 33910562 PMC8082865

[B23] Hiraoka T. Osuga Y. Hirota Y. (2023). Current perspectives on endometrial receptivity: a comprehensive overview of etiology and treatment. J. Obstet. Gynaecol. Res. 49 (10), 2397–2409. 10.1111/jog.15759 37527810

[B24] Izumi G. Koga K. Nagai M. Urata Y. Takamura M. Harada M. (2015). Cyclic stretch augments production of neutrophil chemokines, matrix metalloproteinases, and activin a in human endometrial stromal cells. Am. J. Reprod. Immunol. 73 (6), 501–506. 10.1111/aji.12359 25605062

[B25] Kliman H. J. Frankfurter D. (2019). Clinical approach to recurrent implantation failure: evidence-based evaluation of the endometrium. Fertil. Steril. 111 (4), 618–628. 10.1016/j.fertnstert.2019.02.011 30929719

[B26] Kurmanova G. Ashirbekov Y. Kurmanova A. Mamedaliyeva N. Moshkalova G. Anartayeva G. (2024). Altered expression of C4BPA and CXCL1 genes in the endometrium of patients with recurrent implantation failure after *in vitro* fertilization and thin endometrium. Diagn. (Basel) 14 (17), 1967. 10.3390/diagnostics14171967 PMC11394423 39272751

[B27] Li Q. Wei X. Zeng W. Lin Y. (2025). Impact of IVF-ET on obstetrics and perinatal outcomes in advanced maternal age women. Placenta 167, 55–62. 10.1016/j.placenta.2025.04.022 40328094

[B28] Ma C. Liu G. Liu W. Xu W. Li H. Piao S. (2021). CXCL1 stimulates decidual angiogenesis *via* the VEGF-A pathway during the first trimester of pregnancy. Mol. Cell. Biochem. 476 (8), 2989–2998. 10.1007/s11010-021-04137-x 33770315

[B29] Ma J. Gao W. Li D. (2022). Recurrent implantation failure: a comprehensive summary from etiology to treatment. Front. Endocrinol. (Lausanne) 13, 1061766. 10.3389/fendo.2022.1061766 36686483 PMC9849692

[B30] Margalioth E. J. Ben-Chetrit A. Gal M. Eldar-Geva T. (2006). Investigation and treatment of repeated implantation failure following IVF-ET. Hum. Reprod. 21 (12), 3036–3043. 10.1093/humrep/del305 16905766

[B31] Mumusoglu S. Polat M. Ozbek I. Y. Bozdag G. Papanikolaou E. G. Esteves S. C. (2021). Preparation of the endometrium for frozen embryo transfer: a systematic review. Front. Endocrinol. (Lausanne) 12, 688237. 10.3389/fendo.2021.688237 34305815 PMC8299049

[B32] Neves A. R. Devesa M. Martínez F. Garcia-Martinez S. Rodriguez I. Polyzos N. P. (2019). What is the clinical impact of the endometrial receptivity array in PGT-A and oocyte donation cycles? J. Assist. Reprod. Genet. 36 (9), 1901–1908. 10.1007/s10815-019-01535-5 31352621 PMC6730969

[B33] Norwitz E. R. Schust D. J. Fisher S. J. (2001). Implantation and the survival of early pregnancy. N. Engl. J. Med. 345 (19), 1400–1408. 10.1056/NEJMra000763 11794174

[B34] Noyes R. W. Hertig A. T. Rock J. (2019). Reprint of: dating the endometrial biopsy. Fertil. Steril. 112 (4 Suppl. 1), e93–e115. 10.1016/j.fertnstert.2019.08.079 31623748

[B35] Ohara Y. Matsubayashi H. Suzuki Y. Takaya Y. Yamaguchi K. Doshida M. (2022). Clinical relevance of a newly developed endometrial receptivity test for patients with recurrent implantation failure in Japan. Reprod. Med. Biol. 21 (1), e12444. 10.1002/rmb2.12444 35386362 PMC8967283

[B36] Pathare A. D. S. Loid M. Saare M. Gidlöf S. B. Zamani Esteki M. Acharya G. (2023). Endometrial receptivity in women of advanced age: an underrated factor in infertility. Hum. Reprod. Update 29 (6), 773–793. 10.1093/humupd/dmad019 37468438 PMC10628506

[B37] Robertson S. A. Moldenhauer L. M. Green E. S. Care A. S. Hull M. L. (2022). Immune determinants of endometrial receptivity: a biological perspective. Fertil. Steril. 117 (6), 1107–1120. 10.1016/j.fertnstert.2022.04.023 35618356

[B38] Ruiz-Alonso M. (2021). Endometrial receptivity analysis (ERA): data *versus* opinions. Hum. Reprod. Open 2021 (2), hoab011. 10.1093/hropen/hoab011 33880420 PMC8045472

[B39] Saxtorph M. H. Hallager T. Persson G. Petersen K. B. Eriksen J. O. Larsen L. G. (2020). Assessing endometrial receptivity after recurrent implantation failure: a prospective controlled cohort study. Reprod. Biomed. Online 41 (6), 998–1006. 10.1016/j.rbmo.2020.08.015 32978074

[B40] Sehring J. Beltsos A. Jeelani R. (2022). Human implantation: the complex interplay between endometrial receptivity, inflammation, and the microbiome. Placenta 117, 179–186. 10.1016/j.placenta.2021.12.015 34929458

[B41] Shapiro B. S. Daneshmand S. T. Desai J. Garner F. C. Aguirre M. Hudson C. (2016). The risk of embryo-endometrium asynchrony increases with maternal age after ovarian stimulation and IVF. Reprod. Biomed. Online 33 (1), 50–55. 10.1016/j.rbmo.2016.04.008 27178763

[B42] Shi C. Han H. J. Fan L. J. Guan J. Zheng X. B. Chen X. (2018). Diverse endometrial mRNA signatures during the window of implantation in patients with repeated implantation failure. Hum. Fertil. (Camb) 21 (3), 183–194. 10.1080/14647273.2017.1324180 28523980

[B43] Thota C. S. Reed L. C. Yallampalli C. (2005). Effects of parathyroid hormone like hormone (PTHLH) antagonist, PTHLH(7-34), on fetoplacental development and growth during midgestation in rats. Biol. Reprod. 73 (6), 1191–1198. 10.1095/biolreprod.105.044628 16093356

[B44] Ubaldi F. M. Cimadomo D. Vaiarelli A. Fabozzi G. Venturella R. Maggiulli R. (2019). Advanced maternal age in IVF: still a challenge? The present and the future of its treatment. Front. Endocrinol. (Lausanne) 10, 94. 10.3389/fendo.2019.00094 30842755 PMC6391863

[B45] Wang C. Feng Y. Zhou W. J. Cheng Z. J. Jiang M. Y. Zhou Y. (2021). Screening and identification of endometrial proteins as novel potential biomarkers for repeated implantation failure. PeerJ 9, e11009. 10.7717/peerj.11009 33763303 PMC7958897

[B46] Xu S. (2025). The study on the clinical efficacy of endometrial receptivity analysis and influence factors of displaced window of implantation. Sci. Rep. 15 (1), 7326. 10.1038/s41598-025-91745-y 40025209 PMC11873133

[B47] Yaron Y. Botchan A. Amit A. Kogosowski A. Yovel I. Lessing J. B. (1993). Endometrial receptivity: the age-related decline in pregnancy rates and the effect of ovarian function. Fertil. Steril. 60 (2), 314–318. 10.1016/s0015-0282(16)56104-4 8339830

[B48] Zhao J. (2023). Relationship between advanced maternal age and decline of endometrial receptivity: a systematic review and meta-analysis. Aging (Albany NY) 15 (7), 2460–2472. 10.18632/aging.204555 37036802 PMC10120912

