# Supplementary material for: Advanced maternal age as a risk factor for endometrial receptivity in repeated implantation failure: intersectional transcriptomic analysis and gene validation
Source: Front Cell Dev Biol. 2026 Aug 26;14:1904991. doi: 10.3389/fcell.2026.1904991 (PMC13553768; doi:10.3389/fcell.2026.1904991)
Supplement: Supplementary file 1 [file Table1.docx]

| Primer | Sequence（5’ to 3’） |
| --- | --- |
| ATF3-F | CCTCTGCGCTGGAATCAGTC |
| ATF3-R | TTCTTTCTCGTCGCCTCTTTTT |
| SDK2-F | AGCTCTGCATCAGATCCGC |
| SDK2-R | TGTTGTGGAGCCACTTGAACT |
| CXCL1-F | TCCTCTCACAGCCGCCA |
| CXCL1 -R | ACGGACGCTCCTGCTGC |
| CXCL2-F | CTTGCCAGCTCTCCTCCTC |
| CXCL2-R | AGGGGCGCTCCTGCT |
| PTHLH-F | ATTTACGGCGACGATTCTTCC |
| PTHLH-R | GCTTGGAGTTAGGGGACACC |
| CYP24A1-F | GGTGGCGAGACTCAGAACG |
| CYP24A1-R | GTCGTGCTGTTTCTTGAGACC |
| CHP2-F | GGGGACAGTATTCGGCGAG |
| CHP2-R | TCATCTTCTACAGGGCGAAAATG |
| NCCRP1-F | ATTTCCGTGGCTGGTACATTAG |
| NCCRP1-R | ATGGCTGGTTGTTCGTCATCC |
| GAPDH-F | AAGCTCATTTCCTGGTATGACAA |
| GAPDH-R | CTTACTCCTTGGAGGCCATGT |

Supplementary Table 1. Primer sequence.
