# Supplementary material for: Advanced maternal age as a risk factor for endometrial receptivity in repeated implantation failure: intersectional transcriptomic analysis and gene validation
Source: Front Cell Dev Biol. 2026 Aug 26;14:1904991. doi: 10.3389/fcell.2026.1904991 (PMC13553768; doi:10.3389/fcell.2026.1904991)
Supplement: Supplementary file 2 [file Table2.docx]

Supplementary Table2. Implantation outcomes between young and aged groups following personalized embryo transfer

| Terms | ≤ 30 | > 35 | *P* |
| --- | --- | --- | --- |
|  | (n=21) | (n=30) |  |
| Age ^a^ | 28.6±1.2 | 38.3±2.1 | - |
| Body mass index ^a^ | 23.2±3.1 | 21.5±4.2 | 0.130 |
| Two-day displaced or not ^c^ |  |  | 0.083 |
| normal | 19 (90.5) | 21 (70) | - |
| abnormal | 2 (9.5) | 9 (30) | - |
| bFSH ^b^ | 6.0 (4.9-8.6) | 8.3 (7.3-9.5) | 0.010 |
| bLH ^b^ | 3.9 (3.2-5.2) | 4.5 (3.3-5.5) | 0.681 |
| basal estradiol ^b^ | 32.4 (24.0-42.0) | 47.5 (28.0-67.0) | 0.049 |
| AMH ^b^ | 4.2 (3.0-9.2) | 2.4 (1.5-4.2) | 0.004 |
| Parity ^b^ | 0 (0-1) | 0 (0-1) | 0.177 |
| Endometrial thickness ^a^ | 8.9±1.6 | 8.4±1.7 | 0.278 |
| Early miscarriage rate * | 1 (4.8) | 1 (3.3) | 1.000 |
| Clinical pregnancy rate ^c^ | 10 (47.6) | 12 (40) | 0.649 |
| Implantation rate ^c^ | 12 (34.3) | 12 (27.9) | 0.568 |

a: Data are expressed as mean ± SD for continuous variables; b: Data are expressed as median (first quartile, third quartile) for continuous variables not normally distributed; c: Data are expressed as numbers (percentages) for categorical variables. * Fisher’s Exact Test.

bFSH: basal follicle-stimulating hormone; bLH: basal luteinizing hormone; AMH: anti-Müllerian hormone.
